# Deciphering the Association between *Campylobacter* Colonization and Microbiota Composition in the Intestine of Commercial Broilers

**DOI:** 10.3390/microorganisms11071724

**Published:** 2023-06-30

**Authors:** Jinji Pang, Torey Looft, Qijing Zhang, Orhan Sahin

**Affiliations:** 1 Department of Veterinary Microbiology and Preventive Medicine, College of Veterinary Medicine, Iowa State University, Ames, IA 50011, USA; pjj0702@iastate.edu (J.P.); torey.looft@usda.gov (T.L.); zhang123@iastate.edu (Q.Z.); 2 National Animal Disease Center, United States Department of Agriculture, Ames, IA 50010, USA; 3 Department of Veterinary Diagnostic and Production Animal Medicine, College of Veterinary Medicine, Iowa State University, Ames, IA 50011, USA

**Keywords:** *Campylobacter*, broilers, poultry farm, gut microbiota

## Abstract

*Campylobacter* is a major food safety concern and is transmitted mainly via poultry meat. We previously found that some commercial broiler farms consistently produced *Campylobacter*-negative flocks while others were consistently *Campylobacter*-positive for consecutive production cycles although the farms operated under similar management practices. We hypothesized that this difference in *Campylobacter* colonization might be associated with the gut microbiota composition. To address this, six commercial broiler farms were selected based on their *Campylobacter* status (three negative and three positive) to evaluate the microbiota differences between each farm category. For each farm on each production cycle (2–3 cycles), 40 ceca collected from five-week-old broilers were processed for microbiota analysis via 16S rRNA gene sequencing. Cecal microbiota species richness, phylogenetic diversity, community structure, and composition of *Campylobacter*-positive farms were noticeably different from those of *Campylobacter*-negative farms. *Rikenella*, *Methanocorpusculum*, *Barnesiella*, *Parasutterella*, and *Helicobacter* were significantly more abundant among *Campylobacter*-positive farms. In contrast, *Ruminococcaceae*, *Streptococcus*, *Escherichia*, *Eggerthellaceae*, *Lactobacillus*, *Monoglobus*, and *Blausia* were more abundant in *Campylobacter*-negative farms. *Eggerthellaceae*, *Clostridia*, *Lachnospiraceae*, *Lactobacillus*, *Monoglobus*, and *Parabacteroides* were significantly negatively correlated with *Campylobacter* abundance. These findings suggest that specific members of cecal microbiota may influence *Campylobacter* colonization in commercial broilers and may be further explored to control *Campylobacter* in poultry.

## 1. Introduction

*Campylobacter* is a leading cause of bacterial foodborne gastroenteritis and is a major public health problem worldwide [1]. According to the United States Centers for Disease Control and Prevention (CDC), *Campylobacter* causes an estimated 1.5 million illnesses each year in the United States [2]. A recent report from the Foodborne Diseases Active Surveillance Network (FoodNet) indicated that during 2021 the incidence of infection with foodborne pathogens was highest for *Campylobacter*, which was 17.8 per 100,000 population in 10 monitored cities covering approximately 15% of the U.S. population [3]. Interestingly, compared with the 2016–2018 data, the incidence of *Campylobacter* infection in 2021 decreased by 5.5%, which might be due to the COVID-19 pandemic control measures imposed [3]. *Campylobacter* infection usually causes abdominal cramps and fever; however, bloody diarrhea and vomiting can also occur [4]. An estimated 10% of *Campylobacter* cases may result in hospitalization with a median of three days [5]. *Campylobacter* infections can also occasionally cause severe complications, such as Guillain-Barré syndrome, bacteremia, temporary paralysis, and arthritis, which are sometimes life-threatening [6].

As a major foodborne pathogen, *Campylobacter* can transmit to humans through a variety of routes, such as consumption of contaminated poultry and meat products, unpasteurized milk, and water, as well as contact with pets [7,8]. Contaminated poultry (mainly broiler meat) is considered the main source of human campylobacteriosis [9]. Many studies around the world reported the predominant role of contaminated poultry products in human *Campylobacter* infections using epidemiological and molecular approaches [9,10,11]. Thermophilic *Campylobacter* species, primarily *Campylobacter jejuni* and *Campylobacter coli*, frequently colonize the intestinal tracts of domestic poultry, including chickens, turkeys, ducks, and geese [12,13]. Prevalence studies conducted in Europe, the U.S., and Asia showed that the incidence rate of *Campylobacter* on broiler farms varies from 30% to 100% [14,15,16,17]. During a surveillance study conducted between 2011 to 2012 in the Netherlands, cecal samples obtained from 32 flocks originating from three distinct broiler farms were examined, and the results showed that 43.75% of the flocks tested positive for *Campylobacter* [16]. Another study conducted in 2019 in Mississippi, U.S., found that the overall prevalence of *Campylobacter* from broiler farm samples (litter, feces, and cloacal swab) was 18.5% [18]. The high prevalence of *Campylobacter* on poultry farms is likely to result in an equivalent high contamination rate of *Campylobacter* in retail chicken. According to a recent systematic review and meta-analysis publication, the prevalence of *Campylobacter* in retail broiler chicken in the United States was found to be 59.2% and 55.4% for conventional and alternative production types, respectively [19]. A study conducted across three Australian States from 2016 to 2018 revealed the presence of *Campylobacter* spp. in 90% of retail chicken meat and 73% of chicken offal samples collected [20]. Two studies conducted between 2009 to 2011 in Ireland and Poland showed that the overall prevalence of *Campylobacter* in retail chicken was 84.3% and 50.2%, respectively [21,22].

Controlling *Campylobacter* at the source (i.e., poultry) is important because reducing the prevalence of *Campylobacter* in poultry will also reduce the risk of transmission of *Campylobacter* from poultry to humans. Multiple biosecurity-based on-farm interventions have been proposed to control *Campylobacter* on poultry farms; however, even the most stringent biosecurity measures do not always have a consistent and predictable effect on controlling *Campylobacter* and their effectiveness on controlling flock prevalence is difficult to assess under commercial settings [23,24]. Several non-biosecurity interventions, including the use of vaccines, bacteriocins, probiotics, prebiotics, symbiotics, organic acids, and quorum-sensing inhibitors have been assessed and some demonstrated encouraging outcomes by enhancing gut health and competitively reducing the intestinal *Campylobacter* loads in broiler chickens [12,25,26,27,28,29]. However, their adoption in poultry production remains incomplete and there are still no commercially available products for excluding *Campylobacter* from chickens under commercial production conditions [12,25,26,27,29].

The gastrointestinal (GI) microbiota is a rich microbial community that is important for host health. The GI bacteria of poultry are diverse and play a vital role in not only food digestion but also resistance to pathogens and the development of the immune system [30]. The cecum microbiota was estimated to have at least 1000 different species, with very high absolute counts and complexity [31]. Several gut microbiota members have been shown to have an inhibitory effect on *Campylobacter* colonization in chickens. For example, certain *Lactobacillus* species, which are commonly found in the gut of poultry, were shown to reduce *Campylobacter* colonization by producing inhibitory substances such as lactic acid and bacteriocins [32]. In a previous study, a bacteriocin (OR-7) derived from a *Lactobacillus salivarius* strain was shown to display a remarkable inhibitory effect on *C. jejuni* colonization within the intestinal tract of chicken when incorporated into the feed [33]. Similarly, another bacteriocin (SMXD51) purified from the *L. salivarius* SMXD51 strain, which was isolated from the ceca of chickens, demonstrated anti-*Campylobacter* activity when administered to broilers as a fresh culture [32,34]. In a recent study, *E. coli* Nissle 1917 was shown to reduce *Campylobacter* colonization in chickens by increasing *C. jejuni*-specific and total IgA and IgY antibodies in chicken serum [35]. Similarly, it was reported that the supplementation of *Bacillus subtilis* PS-216 spores in drinking water resulted in a decrease in *C. jejuni* colonization in broilers and concurrently improved their weight gain [36]. The suppression of *Campylobacter* colonization in chickens can be accomplished through various mechanisms, such as modulation of the immune system, improvement of gut physiology, competition for attachment sites, and production of antimicrobial agents by the gastrointestinal microbiome [37,38,39,40]. Despite the promising role of probiotics in mitigating *Campylobacter* colonization in poultry, several studies reported mixed outcomes. Robyn et al. observed that an *Enterococcus faecalis* strain demonstrated inhibitory effects against *Campylobacter* growth in vitro with a difference of at least one log [41]. However, in contrast to the in vitro results, in vivo studies using a seeder bird infection model showed no inhibition of cecal colonization in broilers given *E. faecalis* MB 5259 when challenged with *Campylobacter* [42]. Likewise, Mortada et al., demonstrated the in vitro inhibitory activity of supernatants from four commercial probiotic strains (*Enterococcus faecium*, *Bifidobacterium animalis*, *Pediococcus acidilactici* and *Lactobacillus reuteri*) against *C. coli*; however, regular feed supplemented with the multi-species commercial probiotic (PoultryStar ME, Biomin America, Inc., Overland Park, KS, USA) showed no significant effect on the *C. coli* loads in the broiler ceca or on the carcass [43]. Moreover, in vivo intervention attempts in broilers with probiotics such as *Pediococcus acidilactici* and *Saccharomyces cerevisiae boulardii* failed to demonstrate a significant reduction in *Campylobacter* colonization levels compared to untreated control groups [44].

In a longitudinal study conducted by our team during 2012–2014, the prevalence and genetic diversity of *Campylobacter* on 15 commercial broiler chicken farms operated by the same poultry integrator in the U.S. were investigated [45]. The results of that study showed that the overall *Campylobacter* prevalence at the farm level was high, with a staggering 93% prevalence rate [45]. However, it was found that there were substantial variations in the prevalence of *Campylobacter* among the farms monitored. Of utmost significance was the finding that some farms consistently produced flocks that were *Campylobacter*-negative while other farms consistently reared flocks that were *Campylobacter*-positive throughout the entire eight consecutive production cycles [45]. Although the exact reason for the observed stark difference in *Campylobacter* prevalence among different farms is currently unknown, it is likely to be influenced by multiple factors related to farm management, the environment, and the overall bird health. It is also reasonable to assume that there may be a correlation between the intestinal microbiota and *Campylobacter* status, considering the ample amount of growing evidence supporting the role of gut microbiota in broad spectrum of health and disease conditions, including preharvest pathogen colonization in the animal production [30,37,39,46].

The current study was conducted to assess if the gut microbiota differs between the aforementioned *Campylobacter*-positive and *Campylobacter*-negative poultry farms. We hypothesized that the intestinal microbiota influences *Campylobacter* colonization in chickens and its composition differs significantly between each farm category. To test this hypothesis, we selected six commercial broiler farms based on their *Campylobacter* status (three negative and three positive). The ceca were obtained by the farm personnel when the birds were about 5-weeks old for 2–3 consecutive production cycles and shipped overnight to Iowa State University. The fresh cecal contents were processed for *Campylobacter* culture and 16S rRNA gene-sequencing-based microbiota analysis.

## 2. Materials and Methods

### 2.1. Farm Selection

Six commercial broiler farms (arbitrarily coded as BB, CL, DF, BP, KP, WL to protect confidentiality) were selected to evaluate the study objective. The farms used conventional rearing and were located within a radius of about 30 miles in eastern U.S. These six farms had previously been monitored for eight production cycles during 2012–2014 and identified as either consistently positive (BB, BP, KP) or consistently negative (CL, DF, WL) farms with respect to *Campylobacter* colonization [45]. The farms were all part of the same vertically integrated production system, and thus they all sourced the birds from the same hatchery, reared the same breed of broilers, and used the same feed.

### 2.2. Sample Collection

Cecal contents were collected from adult birds (~5 weeks old) during two or three consecutive production cycles from the six farms (three cycles for WL, two cycles for all the other farms) during 2018–2019. At each cycle, 60 whole individual ceca were obtained by the farm personnel from all four houses on each farm (15 samples/house; 60 samples/farm/cycle), thus yielding a total of 780 samples for this study. The cecum of each bird was collected in a sterile Whirl-Pak bag once the birds were euthanized on the farms. The samples were kept in an insulated box with ice packs and shipped overnight to our laboratory at Iowa State University (ISU), where they were processed on the receiving day (see below). It should be pointed out that the birds were euthanized and necropsied by the poultry company personnel following their procedures and protocols in place while the birds were still in the growout houses for the purpose of company’s own unrelated testing. Thus, an institutional animal care and use committee protocol by ISU was not required.

### 2.3. Campylobacter Status Determination of Farms

For each farm on each production cycle, 40 out of the 60 ceca (10/house) were randomly chosen (520 ceca total), and a small amount of the contents was used for *Campylobacter* culture (presence/absence; no quantification performed). Culture was performed on Mueller-Hinton (MH) agar plates with *Campylobacter* growth supplement (SR084E; Oxoid, Basingstoke, UK) and modified Preston *Campylobacter* selective supplement (SR0204E; Oxoid). Plates were incubated at 42 °C under microaerobic condition (85% N_2_, 10% CO_2_, 5% O_2_) for up to 72 h. The presence of *Campylobacter*-like colonies was judged by their typical morphology (e.g., shiny transparent colonies) and confirmed (one colony per sample) by MALDI-TOF MS following the manufacturer’s (Bruker Daltonik, Billerica, MA, USA) instructions.

### 2.4. DNA Extraction, Amplification, and Sequencing

Total DNA was extracted from the contents of the same sets of 40 ceca used for the culture from each farm per production cycle with the Qiagen DNA Isolation Kit using the manufacturer’s protocol (Qiagen, Hilden, Germany). The V4 hypervariable region of the bacterial 16S rRNA gene was amplified and sequenced using the Illumina MiSeq platform (Illumina, San Diego, CA, USA) as previously described [47,48].

### 2.5. Data Analysis

In total, 520 cecal DNA samples were processed for 16S rRNA gene sequence analysis using the QIIME 2 platform [49]. Demultiplexed sequence data were obtained and denoised using the DADA2 method to eliminate noisy sequences, remove chimeric sequences, and cluster similar sequences (≥99% similarity cut-off) into amplicon sequence variants (ASVs) [50]. Taxonomy classification was performed by comparing the ASVs to the SILVA 16S rRNA gene database [51,52]. Alpha diversity and beta diversity, which describe the microbial diversity within a community and between communities, respectively, were analyzed by the QIIME 2 platform. For alpha diversity analysis, observed features index (i.e., ASVs), Pielou’s evenness index, Shannon index, and Faith’s phylogenetic diversity index were utilized. These indexes describe microbial species richness, evenness, and phylogenetic diversity. For beta diversity analysis, principal coordinates analysis was performed using the Bray–Curtis index to demonstrate the microbiota composition difference between *Campylobacter*-positive and *Campylobacter*-negative farms. Analysis of similarity (ANOSIM) was calculated in R using the vegan package [53]. Differential abundance test was completed in R using the ANCOM-BC package [54]. Correlation analysis of dominant genera was undertaken using Pearson’s correlation test. A *p*-value of <0.05 was considered significant for all statistical analyses. The raw 16S rRNA gene sequencing data for this study will be deposited in the NCBI SRA database.

## 3. Results

### 3.1. Campylobacter Status of the Selected Farms

*Campylobacter* colonization status of the six farms tested in the current study (2018–2019) as well as their colonization status in a previous study (2012–2014) are shown in Table 1. Only one positive farm (BB) and one negative farm (WL) that were found to be consistently positive or negative during the earlier study, respectively, still remained the same during the current study. Two previously positive farms (BP and KP) tested negative on both production cycles in the current study. On the other hand, two previously negative farms (CL and DF) tested positive on both production cycles in the present study. Consequently, the current study comprised three positive farms (BB, CL, DF) and three negative farms (BP, KP, WL) (Table 1). Almost all of the tested cecal contents (n = 236) from the positive farms were *Campylobacter*-positive, except for four samples from farm BB and one from farm CL on a single production cycle, which were negative (Table 1). Close to 100% of the isolates from the positive samples were identified as *C. jejuni.* None of the tested cecal samples (n = 280) from the negative farms yielded *Campylobacter* on culture (Table 1). 

### 3.2. Microbial Diversity

#### 3.2.1. 16S rRNA Gene Sequencing Outputs Overview

In total, 8,624,396 sequence reads and 7889 amplicon sequence variants (ASVs) were obtained from 520 samples on the Illumina MiSeq platform. After filtering out samples with low reads and removing rare features and those belonging to the *Cyanobacteria* phylum, there were 2224 unique features (i.e., ASVs) left from 422 cecal samples for the 16S rRNA gene sequence analysis. The average read frequency per sample was 17,093, which was sufficient to reveal all the features based on the alpha rarefaction result.

#### 3.2.2. *Campylobacter*-Positive Farms Had Higher Cecal Microbiota Species Richness and Phylogenetic Diversity Compared with *Campylobacter*-Negative Farms

**Figure 1:**
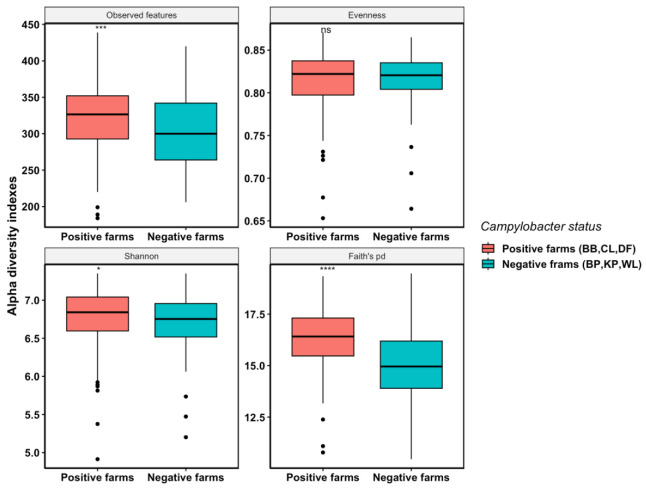
Boxplots for the observed features (ASVs), Pielou’s evenness index, Shannon index, and Faith’s phylogenetic diversity index comparing the microbial diversity of cecal samples collected from three *Campylobacter*-positive farms and three *Campylobacter*-negative farms. The median value is shown as a horizontal line within the box. The inter-quartile range (from lower to upper quartile) represents the middle 50% of values for each group. The *p*-values were calculated using Kruskal–Wallis test (**** *p*-value < 0.0001, *** *p*-value < 0.001, * *p*-value < 0.05, ns: not significant).

Overall, cecal microbial alpha diversities (microbial richness and phylogenetic diversity) were significantly higher among the *Campylobacter*-positive farms (mean observed features = 321; mean Shannon index = 6.77; mean Faith’s pd = 16.29) than those of the *Campylobacter*-negative farms (mean observed features = 303; mean Shannon index = 6.73; mean Faith’s pd = 14.99). *Campylobacter*-positive farms had higher observed features (ASVs) (*p*-value < 0.001), Shannon index (*p*-value < 0.05), and Faith’s phylogenetic diversity index (*p*-value < 0.0001) compared with *Campylobacter*-negative farms, indicating higher species richness and phylogenetic diversity in the cecal microbiome of the positive farms (Figure 1). However, the microbial species evenness was similar between the two populations as no significant differences were observed in the Pielou’s evenness index (Figure 1).

#### 3.2.3. Multiple Factors Contribute to Cecal Microbiota Composition Differences among Farms

**Figure 2:**
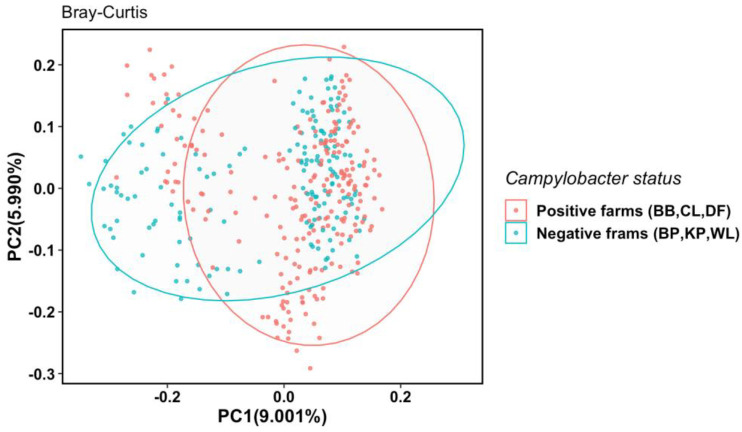
Principal coordinates analysis (PCoA) of cecal microbiota samples from *Campylobacter*-positive (red dots) and *Campylobacter*-negative farms (blue dots) based on the Bray–Curtis dissimilarity index. The PC1 axis depicts 9.001% of the total variance, and the PC2 axis shows 5.990% of the total variance. Each dot represents microbiota composition in a single sample. Each ellipse was drawn at a 95% confidence level to include most samples in either group.

To compare the between-group microbial diversity, principal coordinates analysis (PCoA) was conducted based on Bray–Curtis distances between samples from *Campylobacter*-positive farms and *Campylobacter*-negative farms (Figure 2). Despite a partial overlap, cecal samples from the farms with the same *Campylobacter* status were mostly clustered together, indicating that the cecal microbiota composition of *Campylobacter*-positive farms was distinct from that of *Campylobacter*-negative farms (*p* = 0.001, PERMANOVA test).

**Figure 3:**
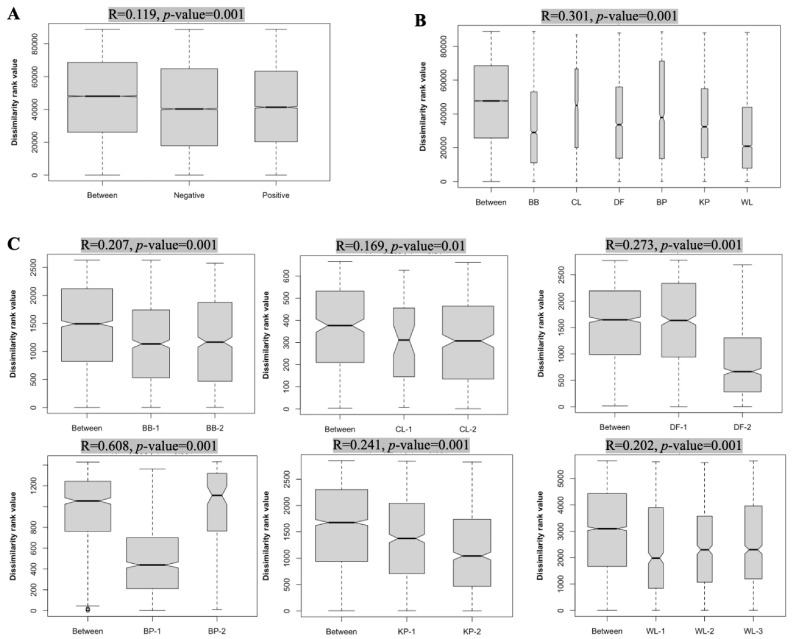
Boxplots for the analysis of similarity (ANOSIM) of the cecal microbiota compositions. ANOSIM results were presented where the bacterial communities were grouped by farm *Campylobacter* status (**A**), farm ID (**B**), and production cycle (**C**). The analysis was conducted using a Bray–Curtis dissimilarity index based on the ASV composition of the total samples within each unit shown on the *x*-axis. The dissimilarity rank values calculated based on 999 permutations between and within groups are shown on the *y*-axis. In (**A**), “Between” indicates the compositional dissimilarities between the *Campylobacter*-negative farms and the positive farms. In (**B**), “Between” indicates the compositional dissimilarities among all of the six farms. In (**C**), “Between” indicates the compositional dissimilarities between the production cycles for each farm. The test results are represented by an R value and the test significance value (*p*-value). An R value close to “1” suggests strong dissimilarity between groups while an R value close to “0” suggests less dissimilarity between groups. A *p*-value less than 0.05 is generally considered to be statistically significant. Negative farms include BP, KP, and WL; positive farms include BB, CL, and DF.

Analysis of similarities (ANOSIM) was conducted to cross-validate the PCoA analysis result and to further illustrate the source of dissimilarity between the two populations. The results indicated that farm *Campylobacter* status, farm ID, and production cycle for a given farm all had significant contributions to the dissimilarity in the cecal microbiotas among the farms (Figure 3). Overall, the *Campylobacter* status of farms (i.e., negative vs. positive) had a moderate but very significant effect on the microbiota composition (Figure 3A). Farm ID and production cycle both had a larger effect on cecal microbiota composition dissimilarity than the farm *Campylobacter* status as revealed by their higher R values (Figure 3).

### 3.3. Cecal Microbiota Overall Was Dominated by Similar Taxa between Campylobacter-Positive and -Negative Farms

**Figure 4:**
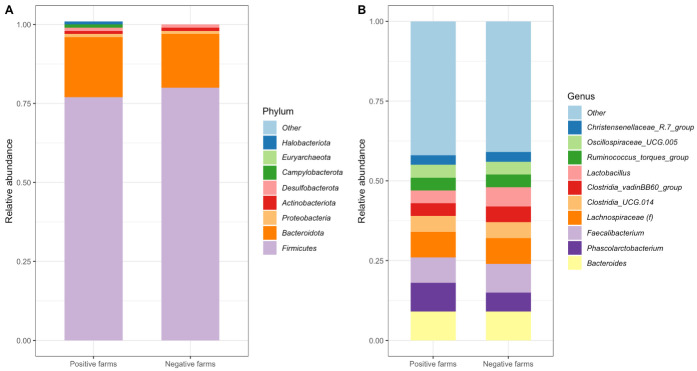
Stacked bar-plot representations of the relative abundances at the phylum (**A**) and genus level (**B**) in *Campylobacter*-positive and *Campylobacter*-negative farms. Phyla that had a relative abundance below 0.1% were aggregated and are shown as Other in the plot, whereas genera that had a relative abundance below 3.3% were treated similarly.

In total, 15 phyla were identified among the six farms studied. The relative frequency of the most dominant phyla in the cecal microbiota of *Campylobacter*-positive and *Campylobacter*-negative farms is shown in Figure 4A. The most abundant phylum from *Campylobacter*-positive farms was *Firmicutes*, representing 76.68% of the sequences, which was followed by *Bacteroidota* (18.66%) and *Proteobacteria* (1.32%). Other major phyla in *Campylobacter*-positive farms with a relative abundance of >0.5% were *Halobacteriota* (0.96%), *Desulfobacterota* (0.71%), *Actinobacteriota* (0.61%), and *Campylobacterota* (0.53%). Similar to *Campylobacter*-positive farms, *Firmicutes* (80.18%) was the most abundant phylum, followed by *Bacteroidota* (17.28%) and *Proteobacteria* (0.99%), in *Campylobacter*-negative farms. Other phyla in *Campylobacter*-negative farms with a relative abundance of >0.5% were *Actinobacteriota* (0.66%) and *Desulfobacterota* (0.52%) (Figure 4A).

At the genus level, a total of 206 taxa were identified among the six farms. The most abundant genus detected in *Campylobacter*-positive farms was *Bacteroides* (9.02%), followed by *Phascolarctobacterium* (8.66%), *Faecalibacterium* (7.75%), and an unknown genus from the *Lachnospiraceae* family (*Lachnospiraceae(f)*, 7.69%) among the genera with relative abundance greater than 5% (Figure 4B). In *Campylobacter*-negative farms, *Faecalibacterium* was the most abundant genus (9.22%), followed by *Bacteroides* (8.94%), *Lachnospiraceae(f)* (7.80%), *Phascolarctobacterium* (5.85%), *Lactobacillus* (5.56%), *and Clostridia_UCG.014* (5.11%) among the genera with a relative abundance of >5% (Figure 4B).

### 3.4. Differentially Abundant Taxa between Campylobacter-Positive and -Negative Farms as Revealed by ANCOM and Correlation Tests

**Figure 5:**
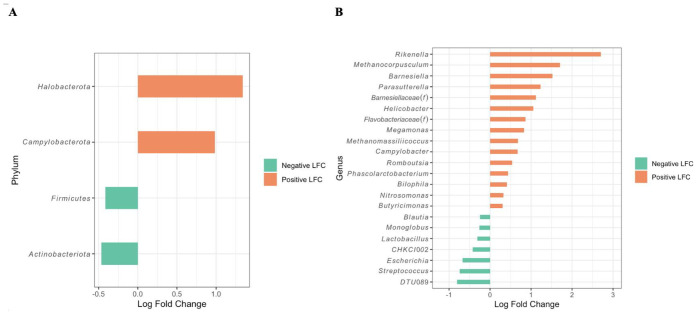
Bar plot of differentially abundant phyla (**A**) and genera (**B**) obtained from ANCOM-BC analysis. Data are represented by log-fold change (LFC) on the *x*-axis. A positive LFC (orange color) indicates that the corresponding taxon is more abundant in *Campylobacter*-positive farms compared with *Campylobacter*-negative farms. A negative LFC (green color) indicates that the corresponding taxon is more abundant in *Campylobacter*-negative farms compared with *Campylobacter*-positive farms. All phyla and genera listed in the figure had a significant LFC with *p*-value < 0.001. CHKCI002: *Eggerthellaceae_CHKCI002*; DTU089: *Ruminococcaceae_DTU 089*.

Even though the overall cecal microbiota compositions between the *Campylobacter*-positive and *Campylobacter*-negative farms were highly similar, as described above, significant differences were detected in the relative abundance of several taxa found in both farm categories as determined by the ANCOM-BC test. At the phylum level, *Halobacteriota* (an *Archaea*) and *Campylobacterota* were significantly more abundant in the samples from *Campylobacter*-positive farms (*Halobacteriota* 0.96%; *Campylobacterota* 0.529%) than those from *Campylobacter*-negative farms (*Halobacteriota* 0%; *Campylobacterota* 0.195%) (Figure 5A). On the other hand, *Firmicutes* and *Actinobacteriota* were significantly more abundant in *Campylobacter*-negative farms (*Firmicutes* 80.18%; *Actinobacteriota* 0.661%) than *Campylobacter*-positive farms (*Firmicutes* 76.68%; *Actinobacteriota* 0.606%). At the genus level, *Rikenella*, *Methanocorpusculum*, *Barnesiella*, *Parasutterella*, *Barnesiellaceae(f)*, *Helicobacter*, and several others, had a log-fold change (LFC) greater than 0, indicating they were significantly more abundant in *Campylobacter*-positive farms than the negative farms (Figure 5B). Conversely, significantly higher abundances of genera *Ruminococcaceae_DTU 089*, *Streptococcus*, *Escherichia*, *Eggerthellaceae_CHKCI002*, *Lactobacillus*, *Monoglobus*, and *Blausia* were detected in *Campylobacter*-negative farms compared with the positive farms (Figure 5B).

**Figure 6:**
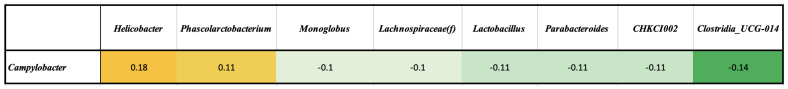
Heat map showing Pearson’s correlation coefficients for *Campylobacter* and other predominant genera at the genus level, as determined by analysis of all cecal samples from both *Campylobacter*-positive and -negative farms. Significant correlations are colored in yellow (positive) or green (negative). Only statistically significant (*p*-value < 0.05) correlation coefficients are shown. Coefficients range from −1 to 1, with 1 representing the highest positive correlation and −1 representing the highest negative correlation. CHKCI002: *Eggerthellaceae_CHKCI002*.

The interaction among the most abundant genera from all the cecal samples, including both *Campylobacter*-positive farms and *Campylobacter*-negative farms, was further investigated using Pearson’s correlation. The analysis indicated that the overall *Campylobacter*-positive status of the samples was positively correlated with *Helicobacter* (correlation coefficient = 0.18; *p*-value < 0.05) and *Phascolarctobacterium* (correlation coefficient = 0.11; *p*-value < 0.05), and negatively correlated with *Eggerthellaceae_CHKCI002* (correlation coefficient = −0.11; *p*-value < 0.05), *Clostridia_UCG.014* (correlation coefficient = −0.14; *p*-value < 0.05), *Lachnospiraceae(f)* (correlation coefficient = −0.1; *p*-value < 0.05), *Lactobacillus* (correlation coefficient = −0.11; *p*-value < 0.05), *Monoglobus* (correlation coefficient = −0.1; *p*-value < 0.05), and *Parabacteroides* (correlation coefficient = −0.11, *p*-value < 0.05) (Figure 6).

## 4. Discussion

Investigation of the specific differences in the intestinal microbiota compositions between *Campylobacter*-positive and *Campylobacter*-negative poultry flocks has been a research hotspot for the past decades [55,56]. Although several studies claimed that certain gut microbiota members might be involved in the protection of poultry from *Campylobacter* colonization, most of the identified taxa were at order or family levels and did not reveal any significant genus level differences [57,58]. In the current study, we sought to provide more detailed data at lower taxonomic levels (e.g., genus) in order to better determine if there was a tangible association between *Campylobacter* colonization and microbiota composition in the intestine of commercial broiler chickens. The preliminary foundation of this study was from a previous longitudinal study conducted by our research team which found a considerable variation in *Campylobacter* prevalence among different commercial broiler farms that were managed by the same company (i.e., the chicks were sourced from the same hatchery and consumed the same type of feed) [45]. Specifically, some farms consistently produced *Campylobacter*-negative flocks while other farms consistently produced *Campylobacter*-positive flocks during the entire surveillance period (i.e., eight consecutive production cycles) [45]. The present study analyzed the cecal microbiota compositions of adult broiler chickens raised on farms with different *Campylobacter* statuses during multiple production cycles. We observed that the cecal microbiota diversity was significantly higher for *Campylobacter*-positive farms compared with *Campylobacter*-negative farms. Additionally, both the composition and the structure of cecal microbiota exhibited significant differences between farms with different *Campylobacter* statuses. A key finding of this study is that we identified several genera (i.e., *Lactobacillus*, *Eggerthellaceae_CHKCI002*, and *Monoglobus*) that were significantly more abundant in the microbiota of *Campylobacter*-negative flocks than that of *Campylobacter*-negative flocks, as revealed by both differential abundant test and correlation analysis (Figure 5 and Figure 6).

As the first step of the current study, the *Campylobacter* status of the commercial broiler farms that reared consistently positive (n = 3) or negative (n = 3) flocks, as determined in our previous study performed during 2012–2014, was reevaluated in 2018–2019. Unfortunately, albeit not too surprisingly, only one farm from each category retained their previous status during the later testing period (Table 1). Although this highlights the dynamic nature of *Campylobacter* status, we were still able to identify two more farms from each category with consistently negative or positive *Campylobacter* status over two–three production cycles. In total, three *Campylobacter*-positive and three *Campylobacter*-negative farms (each with four separate houses) were included in the present study, thus providing a large number of cecal samples (n = 520) to assess the association between farm *Campylobacter* status and microbiota composition. Although the exact reason(s) for the switch observed in *Campylobacter* status of the farms between two different periods are difficult to ascertain, multiple factors related to the management and environment are likely to be involved.

Interestingly, the cecal microbial richness was found to be significantly higher for the *Campylobacter*-positive farms than for the *Campylobacter*-negative farms in the present study (Figure 1). Generally, it is considered that diverse and balanced gut microbiota can provide a competitive disadvantage for enteric zoonotic bacteria with pathogenic potential (e.g., *Salmonella* and *Campylobacter*), reducing their ability to colonize and persist in the gut, while reduced gut microbiota diversity may provide an advantageous environment for the same zoonotic bacteria to thrive in the intestinal tract, leading to an increased risk of foodborne illness [30,59]. In contrast to this common finding/perception, our results showed that *Campylobacter*-positive farms had significantly higher microbial richness and phylogenetic diversity (Figure 1). In line with our results, the species richness of *Campylobacter*-positive flocks was found to be significantly higher than that of the *Campylobacter*-negative flocks on commercial broiler farms located in Northern Italy [60]. Likewise, another study reported that increased colonization of *Campylobacter* in commercial broilers also led to an increase in microbial richness [61]. In contrast, Sofka et al. detected 55 genera in *Campylobacter*-negative commercial broiler cecal samples and 47 genera in *Campylobacter*-positive cecal samples by 16S rRNA gene sequencing and found a higher cecal microbiota diversity in the former group [62]. In the same study, significantly lower numbers of culturable bacteria, including lactic acid bacteria, *Staphylococci*, *Enterococci*, *Enterobacteriaceae*, and *E. coli*, were also observed in *Campylobacter*-positive samples [62]. In contrast to these findings, Hertogs et al. observed no significant differences in bacterial richness between *Campylobacter*-positive and *Campylobacter*-negative cecal dropping samples of commercial broilers [55]. Another study examined the impact of the exposure time to *C. jejuni* on the gut microbiome of broilers under experimental settings and found that the richness of the cecal microbial communities was typically unaffected by *C. jejuni* colonization between the early and late *Campylobacter* exposure times [57]. 

Chicken gut microbiota diversity and composition can be influenced by many factors, such as breed and age of birds, type of production system, geographical location of farms, type of litter used during the rearing period, and composition of the feed ration consumed [63]. In the current study, all the broiler birds employed were of the same breed and age, given the same feed rations, and produced under comparable management practices, as all the farms were part of the same vertically integrated poultry production system. Therefore, it was reasonable for us to hypothesize that the variation in the cecal microbiota diversity and composition seen in this study might be associated with the *Campylobacter* status of the farms. This was indeed found to be an accurate assumption, as revealed by the ANOSIM test results (Figure 3). It should be pointed out that although both the farm ID (location) and production cycle (repeat sampling of the same farm) had a greater effect on the microbiota composition than the farm *Campylobacter* status, the latter still had a significant impact (*p*-value = 0.001) that should not be overlooked. We believe that the association found between the farm *Campylobacter* status and microbiota composition in this study is highly insightful and strongly supported by the sampling method employed (i.e., analysis of a large number of cecal contents from consistently negative or positive farms over multiple consecutive production cycles). Similar to our observations, Patuzzi et al. reported that both time and farm were factors that had a significant effect on the cecal microbial communities of commercial broiler chickens [60]. Moreover, another study reported a clear separation of cecal microbiota compositions between *Campylobacter*-negative and *Campylobacter*-positive commercial broiler flocks in a three-dimensional PCoA plot [62].

Significant differences in the abundance of specific bacterial taxa at different taxonomic levels between *Campylobacter*-positive farms and *Campylobacter*-negative farms were identified in the current study (Figure 4). Not surprisingly, at phylum level, *Campylobacterota*, which is mainly composed of *Campylobacter* spp. and *Helicobacter* spp., was more abundant in the *Campylobacter*-positive farms compared with the *Campylobacter*-negative farms. Despite slight differences, both farm categories regardless of their *Campylobacter* status shared the identical top three most abundant microbial phyla, i.e., *Firmicutes*, *Bacteroidota*, and *Proteobacteria*. These findings are in agreement with previous investigations that reported *Firmicutes* as the most predominant phylum, followed by *Bacteroidota*, *Proteobacteria*, and *Actinobacteria* in the cecal microbiota of various poultry species [64,65,66]. Although *Firmicutes* was the most abundant phylum in both *Campylobacter*-positive farms and *Campylobacter*-negative farms in our study, its abundance was significantly higher in the latter (Figure 4A and Figure 5A). A similar finding was also reported by another study conducted in Australia, where *Campylobacter*-negative commercial broiler flocks were found to have a higher amount of *Firmicutes* (average 62%) than *Campylobacter*-positive flocks (36.6%) [62]. 

At genus level, greater heterogeneity was observed in the most abundant microbial populations between the two farm categories (Figure 4). Specifically, whereas *Bacteroides* was identified as the predominant genus in the *Campylobacter*-positive population, the *Campylobacter*-negative population was characterized by the predominance of the genus *Faecalibacterium*. *Faecalibacterium* is a member of the *Ruminococcaceae* family and assumes a significant function in anaerobiosis by conveying electrons to oxygen, thereby preserving a strictly anaerobic milieu devoid of alternative electron acceptors, such as nitrogen or sulfate [67]. This characteristic restricts enteric pathogens, such as *E. coli* or *Salmonella*, from exploiting the more efficient anaerobic respiratory metabolism, hence inhibiting their overgrowth [67]. *Faecalibacterium* is also recognized as one of the most ubiquitous bacterial genera in the human gut microbiome, and a paucity of *Faecalibacterium* has been correlated with the occurrence of inflammatory bowel disease [67]. Although the relative abundance of *Faecalibacterium* did not differ significantly between *Campylobacter*-positive and *Campylobacter*-negative populations, its predominance and higher abundance in the latter population suggests a potential association with the *Campylobacter* status of the farms investigated in the present study.

Differential abundant testing (ANCOM-BC) at genus level was conducted to further evaluate the cecal microbiome compositional differences between *Campylobacter*-negative and *Campylobacter*-positive farms (Figure 5B). Considering the significantly differentially abundant genera, the *Campylobacter*-negative farms exhibited a considerably higher abundance of several genera including *Ruminococcaceae_DTU 089*, *Streptococcus*, *Escherichia*, *Eggerthellaceae_CHKCI002*, *Lactobacillus*, *Monoglobus*, and *Blausia*, whereas the *Campylobacter*-positive farms exhibited a considerably higher abundance of several other genera, including *Rikenella*, *Methanocorpusculum*, *Barnesiella*, *Parasutterella*, *Helicobacter*, *Megamonas*, *Methanomassiliicoccus*, *Romboutsia*, *Phascolarctobacterium*, *Bilophila*, *Nitrosomonas*, and *Butyricimonas*. In contrast to these findings from our study, a similar study conducted in Belgium found that *Streptococcus* was significantly more abundant in *Campylobacter*-colonized commercial broiler flocks, while *Megamonas*, *Helicobacter*, and *Barnesiella* were found to be more abundant in *Campylobacter*-free flocks [55]. A possible explanation for this could be that the Belgian study employed cecal dropping samples in the 16S rRNA gene analysis, as opposed to the cecal contents used in our study.

Similar to the ANCOM-BC test, Pearson’s correlation analysis also revealed a negative association between the abundance of *Campylobacter* and various other genera, including *Eggerthellaceae_CHKCI002*, *Clostridia_UCG.014*, *Lachnospiraceae(f)*, *Lactobacillus*, *Monoglobus*, and *Parabacteroides* (Figure 6). The overlapping outcomes of the two statistical analyses indicated *Eggerthellaceae_CHKCI002*, *Lactobacillus*, and *Monoglobus* as being more abundant in *Campylobacter*-negative farms than in *Campylobacter*-positive farms, suggesting that these genera could potentially play an antagonistic role in *Campylobacter* colonization in chickens. A previous study has shown a positive correlation between the genera *Eggerthellaceae_CHKCI002* and *Monoglobus* and butyrate production in poultry [68]. Butyrate is a short-chain fatty acid (SCFA) that maintains intestinal homeostasis through its anti-inflammatory effects [69,70]. Interestingly, Kang et al. reported a lower abundance of *Eggerthellaceae_CHKCI002* in layer chickens challenged experimentally with *Salmonella* Enteritidis compared with the non-challenged *Salmonella*-free birds [71]. *Lactobacillus* is a well-known probiotic and has been extensively evaluated for its anti-*Campylobacter* activity under both in vitro and in vivo conditions [72]. In vitro, several *Lactobacillus* species, including *Lactobacillus johnsonii*, *Lactobacillus reuteri*, *Lactobacillus crispatus*, *Lactobacillus gasseri*, and *Lactobacillus salivarius*, have been found to have both immunomodulatory and anti-*Campylobacter* activities by multiple mechanisms such as reducing the expression of virulence-related genes responsible for motility, invasion, and AI-2 production [73]. A particular strain of *Lactobacillus*, *L. salivarius* SMXD51, was found to produce a type of bacteriocin with broad-spectrum antimicrobial properties, which was shown to inhibit the growth of both Gram-positive and Gram-negative bacteria, including *C. jejuni* and *C. coli*, in vitro [34,74]. Furthermore, feeding broilers periodically with the *L. salivarius* SMXD51 strain was also shown to result in a significant reduction in *Campylobacter* colonization in broilers under laboratory conditions [32]. 

The increased abundance of *Helicobacter* and *Phascolarctobacterium* in the *Campylobacter*-positive farms, as indicated by both statistical analyses (Figure 5 and Figure 6), implies that these two genera may engage in mutualistic interactions with *Campylobacter* within the cecal ecosystem. *Campylobacter* and *Helicobacter* are both Gram-negative microaerophilic bacteria that can cause disease in humans and are often found in the microbiota of chickens and on processed chicken meat [75,76]. Of note, *Helicobacter* were once named *Campylobacter* because of their great similarity to each other [75]. Therefore, it is not entirely surprising to see that the abundance of *Campylobacter* was positively correlated with the abundance of *Helicobacter* in the cecal contents analyzed in this study. *Phascolarctobacterium* has been recognized as a potentially beneficial microorganism owing to its capacity to synthesize short-chain fatty acids such as acetate and propionate [77]. Consequently, it has been incorporated into Aviguard, a commercially available competitive exclusion product, to improve the resistance of chickens to *Salmonella* [31]. The augmentation *Phascolarctobacterium*, along with other probiotic bacteria present in Aviguard such as *Megamonas* and *Parasutterella*, in *Campylobacter*-positive farms observed in the present study was somewhat unexpected. However, Aviguard was primarily designed to target *Salmonella*, and *Salmonella* was not identified in our samples, regardless of farm status. A recent investigation examined the impact of Aviguard on the inflammatory responses of mice challenged with *C. jejuni* strain and observed that administrating Aviguard resulted in an improved clinical outcome and attenuated the apoptotic cell response in the large intestine during acute campylobacteriosis [78]. Interestingly, even though Aviguard resulted in a significant decrease in *C. jejuni* colonization levels in the intestine of broiler chickens in comparison with birds treated with bacitracin, no such effect was observed in untreated control birds in an experimental challenge study [44]. 

In the current study, we assessed the differences in the cecal microbiotas of flocks with and without *Campylobacter* under commercial production conditions. However, it is important to note that previous research has also examined the relationship between the microbiota of broiler litter and the *Campylobacter* isolation status of the flocks. For instance, by examining the microbiome of the broiler litter, two studies discovered that *Bifidobacterium*, *Anaerosporobacter*, *Stenotrophomonas*, *Bogoriella*, and *Pseudogracilibacillus* were substantially and adversely linked with *Campylobacter* abundance in chicken flocks [79,80]. By carrying out a Dynamic Bayesian networks study, Valeris-Chacin et al. also discovered a highly negative association between *Campylobacter* and two bacterial taxa in the broiler littler, *Ornithinibacillus* and *Oceanobacillus* [80]. Although the aforementioned genera found to be negatively associated with *Campylobacter* in broiler litter were not detected in our study, it still supports the hypothesis that certain broiler gut microbes may harbor the potential of disrupting *Campylobacter* colonization in commercial broilers.

The current study has several limitations. First, as mentioned previously, the *Campylobacter* colonization status of the four farms selected for microbiota analysis shifted between the two surveillance periods (2012–2014 and 2018–2019). Even though this was not an entirely unusual or unexpected finding considering the long duration between the two periods, it shows the potential for *Campylobacter* colonization status of farms to change over time. Second, each farm was sampled for only two or three consecutive production cycles and the production cycles (sampling dates) for a given farm tested had a strongly significant effect on the cecal microbiota composition. Increasing the number of production cycles per farm or collecting samples throughout the year may weaken the effect of the sampling date/cycle on the analysis to better dissect the differences in gut microbiota composition between *Campylobacter*-positive and *Campylobacter*-negative farms. Despite these limitations, the current study comprised a large number of cecal samples from multiple farms and houses with consistent *Campylobacter* status over multiple consecutive production cycles, providing enough statistical power for comparative analysis. It should also be pointed out that this study represents one of the few available in the published literature that attempted to delineate the disparity in the cecal microbiota of broiler chickens between *Campylobacter*-positive and *Campylobacter*-negative farms under commercial settings [55,60,62].

## 5. Conclusions

The current study undertook a comparative analysis of the cecal microbiotas of two distinct broiler populations: one harboring *Campylobacter* and the other being free of *Campylobacter*. A noteworthy augmentation was revealed in the microbial diversity within the cecal ecosystems of *Campylobacter*-positive flocks, particularly in the context of increased species richness and phylogenetic diversity. It was further revealed that several genera such as *Rikenella*, *Methanocorpusculum*, *Barnesiella*, *Parasutterella*, *Barnesiellaceae(f)*, and *Helicobacter* had higher abundances in *Campylobacter*-positive farms. In contrast, genera such as *Ruminococcaceae_DTU 089*, *Streptococcus*, *Escherichia*, *Eggerthellaceae_CHKCI002*, *Lactobacillus*, *Monoglobus*, and *Blausia* were more abundant in farms that were free of *Campylobacter.* Through a comprehensive analysis of taxonomic compositions, *Eggerthellaceae_CHKCI002*, *Lactobacillus*, and *Monoglobus* were identified as members of the cecal microbiota that may potentially exhibit an inhibitory effect on *Campylobacter* colonization in broiler chickens. Overall, these findings may be valuable for developing an evidence-based approach to design tailored gut microbial communities that may be used to mitigate *Campylobacter* colonization in broilers, thereby enhancing food safety. For example, our findings can serve as a guide for probiotic development. It may be possible to produce tailored probiotic formulations by identifying specific bacteria that are noticeably more abundant on chicken farms without *Campylobacter*. Reduced incidence of *Campylobacter* colonization in chickens (and thus reduced risk of transmission to humans) may be achieved by using probiotics that can either competitively exclude or inhibit *Campylobacter* in poultry.

## Figures and Tables

**Table 1:** *Campylobacter* colonization status of six broiler farms included in the current study (2018–2019). The colonization status as determined in a previous study (2012–2014) is also shown.

Farm ID *	*Campylobacter* Status (2012–2014)	*Campylobacter* Status (2018–2019)	No. Samples Used in This Study	No. Samples Tested Positive in This Study
BB	Positive	Positive	80	76
CL	Negative	Positive	80	79
DF	Negative	Positive	80	80
BP	Positive	Negative	80	0
KP	Positive	Negative	80	0
WL	Negative	Negative	120	0

* The samples were from two consecutive production cycles for all farms except for farm WL (from three consecutive cycles). Colonization status was determined by culture.

## Data Availability

The 16S rRNA amplicon sequencing data from this study were submitted to the NCBI SRA database under BioProject accession no. PRJNA985591.
